# Supplementary material for: Dendritic Cell Vaccines Impact the Type 2 Innate Lymphoid Cell Population and Their Cytokine Generation in Mice
Source: Vaccines (Basel). 2023 Oct 3;11(10):1559. doi: 10.3390/vaccines11101559 (PMC10610585; doi:10.3390/vaccines11101559)
Supplement: Supplementary file 1 [file vaccines-11-01559-s001.zip › vaccines-2499116-supplementary.pdf]

vaccines

1.a

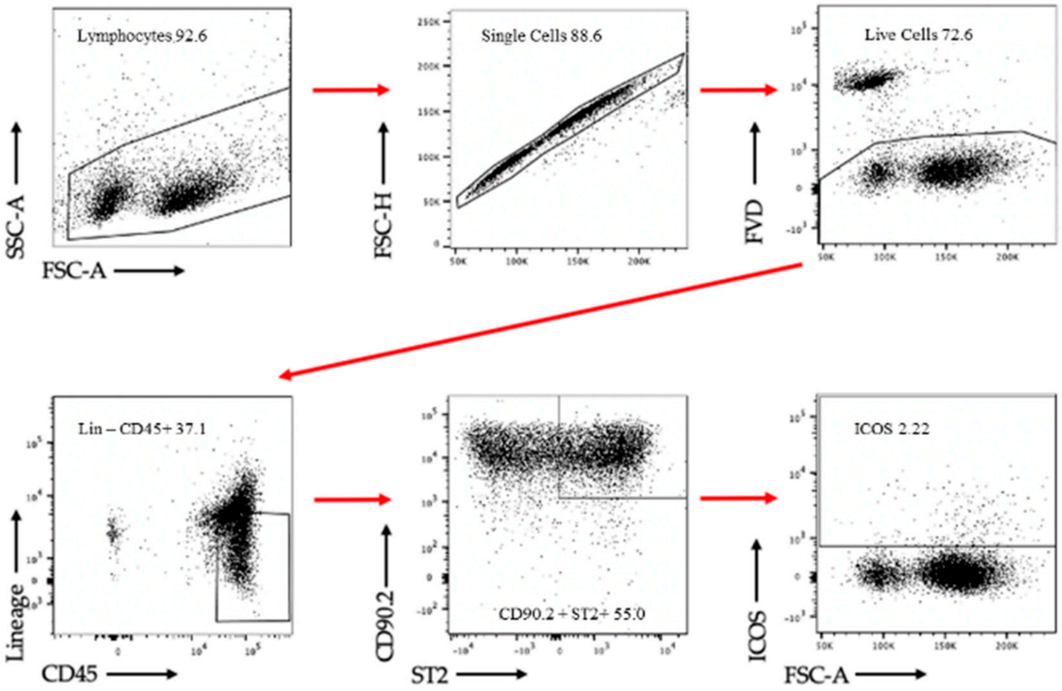

1.b

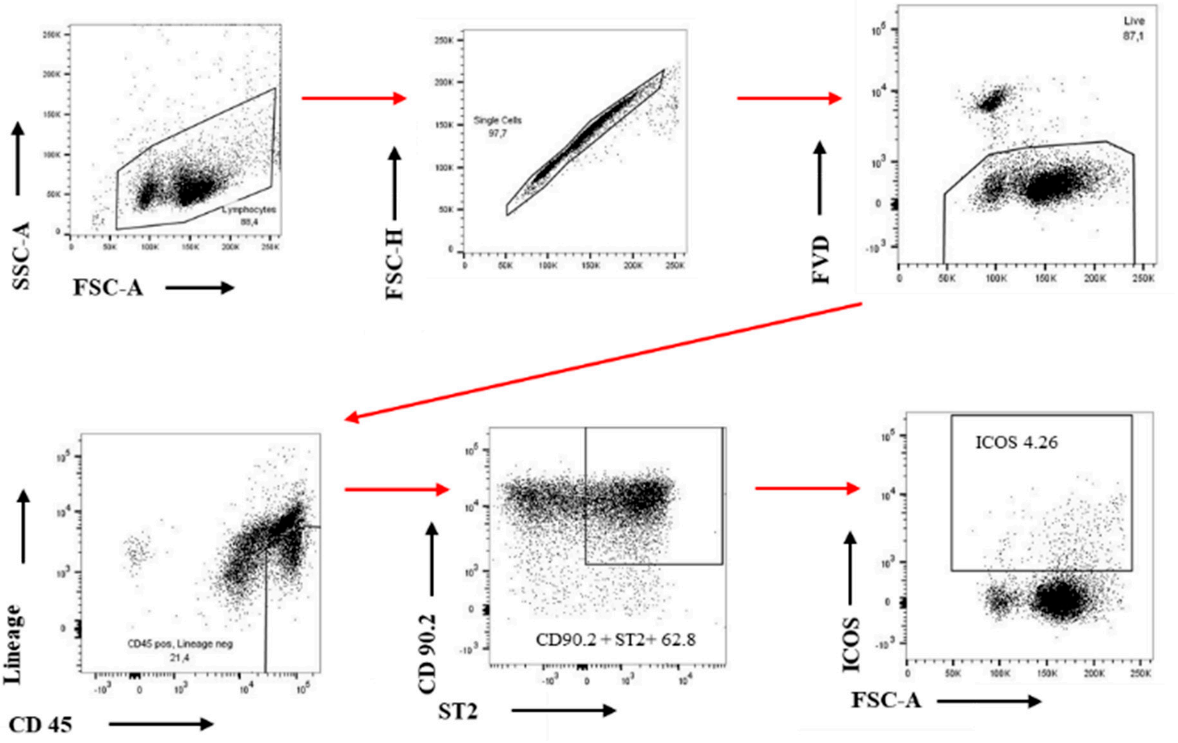

1.c

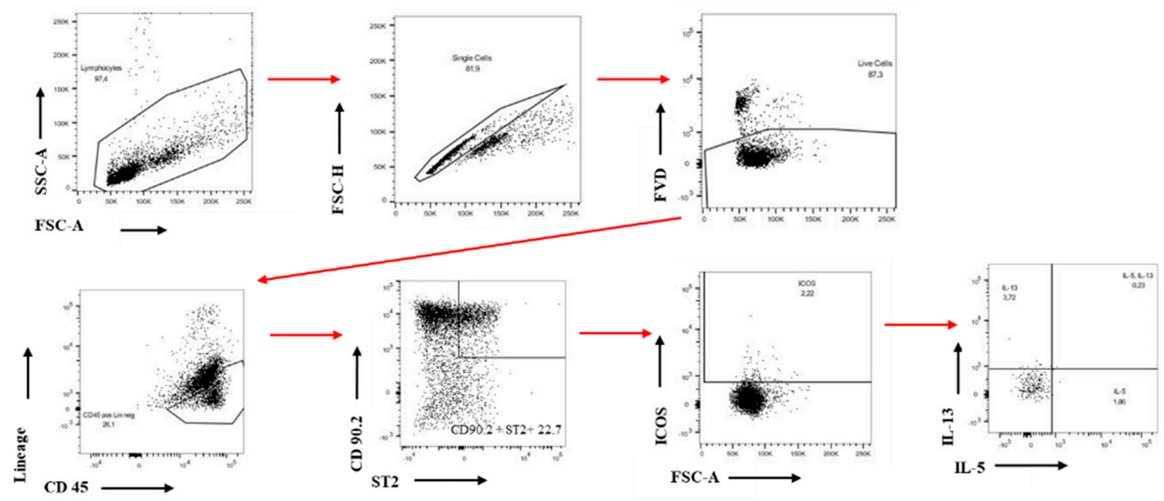

1.d

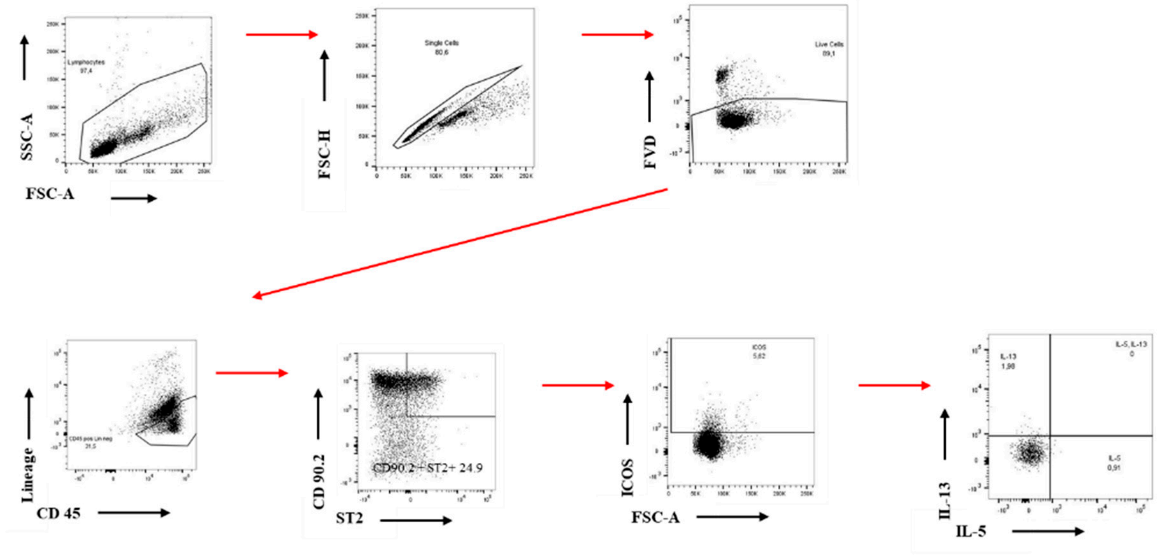

Figure S1. Representative dot plots demonstrating the flow cytometry gating strategies to identify type 2 innate lymphoid cells (ILC2s) in lymph nodes of naïve mice (**a**), DC vaccinated mice (**b**), spleens of naïve mice (**c**), and spleens of DC vaccinated mice (**d**). First, forward scatter-area (FSC-A) and side scatter-area (SSC-A) were used to gate the lymphocyte population. Then, using FSC-A and forward scatter height (FSC-H), doublets were excluded. The live cells were then gated based on fixable viability dye (FVD) negativity. The CD45^{+} and lineage- population was gated from the live cells. Then, the CD90.2^{+} and ST2^{+} population was gated on. From this population, the ICOS^{+} cells were defined as ILC2s. In the spleen, this ILC2 population was then gated on and assessed for IL-13 and IL-5 positivity.

2.a

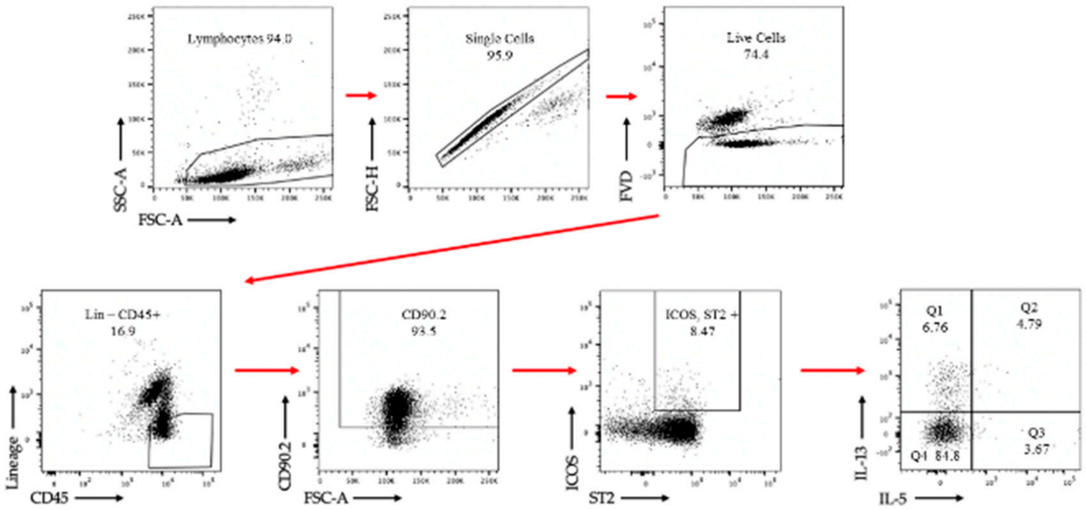
Lymphocytes 94.0
Single Cells 95.9
Live Cells 74.4
SSC-A
FSC-A
FSC-H
FVD
Lin – CD45+ 16.9
CD90.2 93.5
ICOS, ST2 + 8.47
Q1 6.76
Q2 4.79
Q3 3.67
Q4 84.8
Lineage
CD45
CD90.2
ICOS
ST2
IL-13
IL-5

2.b

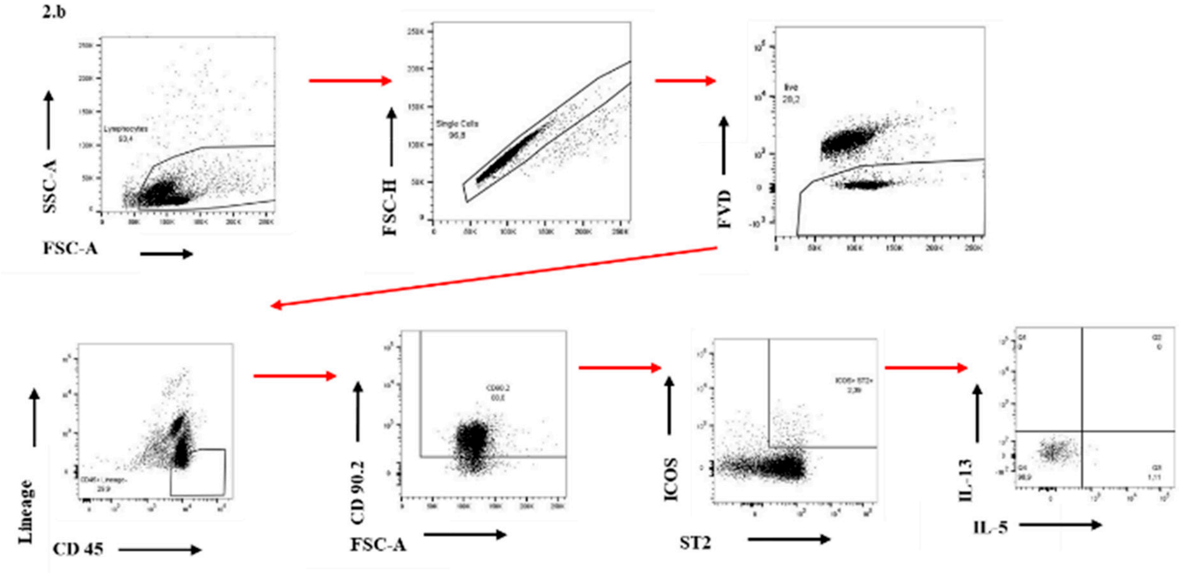
SSC-A
FSC-A
FSC-H
FVD
Lineage
CD 45
CD 90.2
ICOS
ST2
IL-13
IL-5

2.c

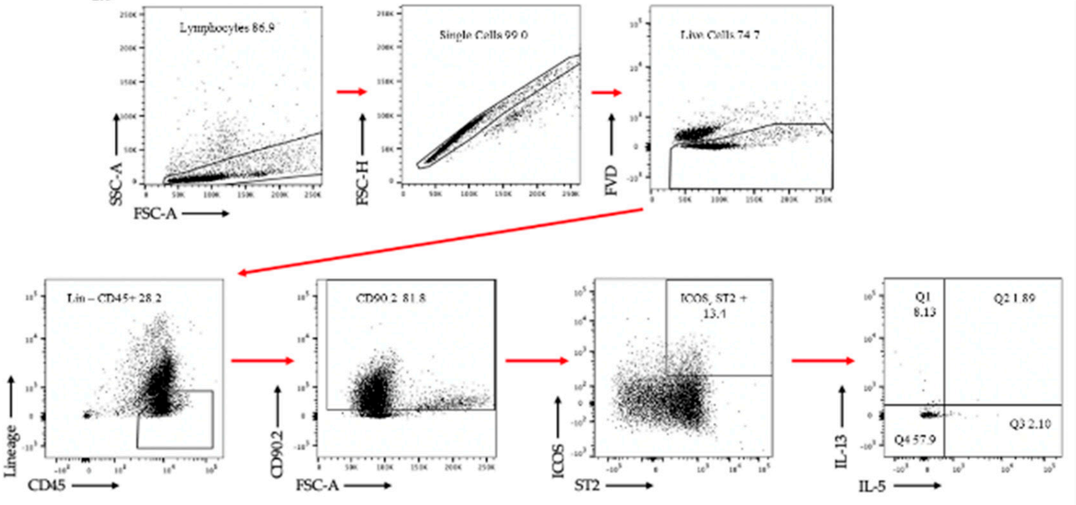
Lymphocytes 86.9
Single Cells 99.0
Live Cells 74.7
SSC-A
FSC-A
FSC-H
FVD
Lin – CD45+ 28.2
CD90.2 81.8
ICOS, ST2 + 13.1
Q1 8.13
Q2 1.89
Q3 2.10
Q4 57.9
Lineage
CD45
CD90.2
ICOS
ST2
IL-13
IL-5

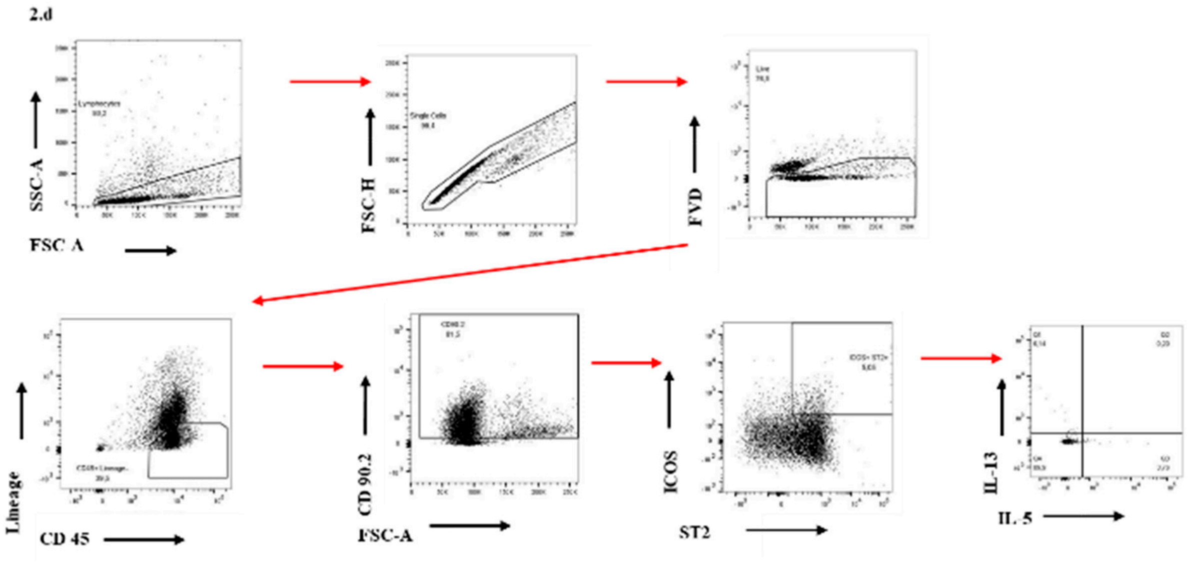

Figure S2. Representative dot plots demonstrating the flow cytometry gating strategies to identify ILC2s in spleens and lungs of tumor-bearing mice treated with PBS (spleen (**a**) and lung (**c**)) or DC-vaccinated (spleen (**b**) and lung (**d**)). Lymphocytes were gated using side scatter-area (SSC-A) by forward scatter area (FSC-A), and then singlet cells were gated using FSC-A and forward scatter-height (FSC-H). The live cells were gated from this population by taking the events negative for fixable viability dye (FVD). Then, the CD45$^+$ and lineage$^-$ population was gated on from the live cells. From this population, CD90.2$^+$ cells were gated on. Then, the ICOS$^+$ and ST2$^+$ populations were defined as ILC2s. The ILC2s were then assessed for IL-5 and IL-13 positivity.

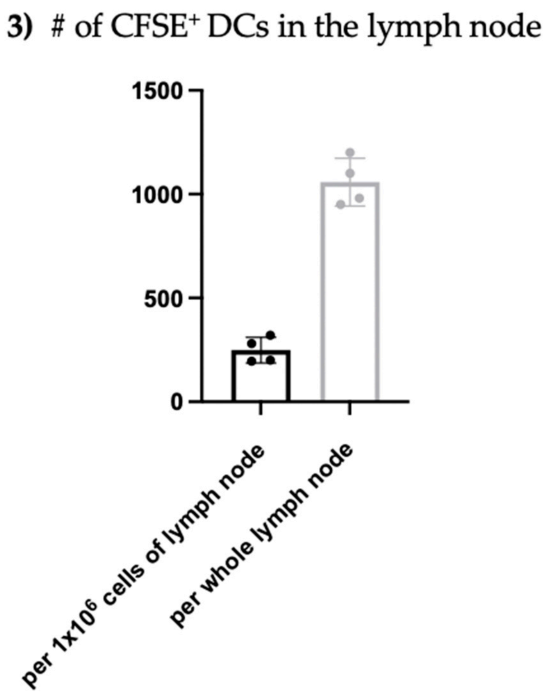

Figure S3. The dendritic cell (DC) vaccine migrated to the local draining lymph node 24 hours post-DC inoculation. DC vaccines were generated in vitro and fluorescently labeled with carboxyfluorescein succinimidyl ester (CFSE) prior to injection into the hind footpads of mice (1x10^6 cells per 30 μL injection dose; totally 4 mice). The popliteal lymph nodes were examined for migration of the DC vaccine from the footpad. The number of migrated DCs (CFSE$^+$ CD11c$^+$ cells) in the whole lymph node per 1x10^6 total cells was determined using flow cytometry analysis 24 hours post-DC vaccination.

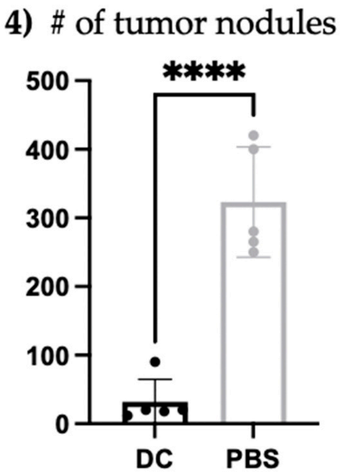

Figure S4. Prophylactic dendritic cell (DC) vaccination limited tumor nodule development in mice. DC vaccines were prepared using in vitro-cultured DCs, and seven to nine-week-old female C57BL/6 mice were immunized with $1x10^6$ DCs or phosphate-buffered saline (PBS) via hind footpad injection. 1×10^6 syngeneic B16F10 melanoma cells were injected intravenously seven days after DC immunization, and mice were euthanized 14 days thereafter. The number of tumor nodules on the lungs was quantified visually and compared between PBS control mice and DC-immunized mice (5 mice per group). Statistical analysis was determined using Student's t-test (****p-value<0.0001).
